# Acellular Tissue-Engineered Vascular Grafts from Polymers: Methods, Achievements, Characterization, and Challenges

**DOI:** 10.3390/polym14224825

**Published:** 2022-11-09

**Authors:** Xinyu Wang, Vincent Chan, Peter R. Corridon

**Affiliations:** 1 Department of Biomedical Engineering and Healthcare Engineering Innovation Center, Khalifa University, Abu Dhabi P.O. Box 127788, United Arab Emirates; 2 Department of Immunology and Physiology, College of Medicine and Health Sciences, Khalifa University, Abu Dhabi P.O. Box 127788, United Arab Emirates; 3 Center for Biotechnology, Khalifa University, Abu Dhabi P.O. Box 127788, United Arab Emirates

**Keywords:** acellular, multiscale, tissue-engineered vascular grafts, polymers, physical and biomechanical characterization

## Abstract

Extensive and permanent damage to the vasculature leading to different pathogenesis calls for developing innovative therapeutics, including drugs, medical devices, and cell therapies. Innovative strategies to engineer bioartificial/biomimetic vessels have been extensively exploited as an effective replacement for vessels that have seriously malfunctioned. However, further studies in polymer chemistry, additive manufacturing, and rapid prototyping are required to generate highly engineered vascular segments that can be effectively integrated into the existing vasculature of patients. One recently developed approach involves designing and fabricating acellular vessel equivalents from novel polymeric materials. This review aims to assess the design criteria, engineering factors, and innovative approaches for the fabrication and characterization of biomimetic macro- and micro-scale vessels. At the same time, the engineering correlation between the physical properties of the polymer and biological functionalities of multiscale acellular vascular segments are thoroughly elucidated. Moreover, several emerging characterization techniques for probing the mechanical properties of tissue-engineered vascular grafts are revealed. Finally, significant challenges to the clinical transformation of the highly promising engineered vessels derived from polymers are identified, and unique perspectives on future research directions are presented.

## 1. Introduction

Vascular malfunctions contribute to various maladies and have emerged as a leading cause of global mortality. Specifically, as much as 32% of deaths worldwide were attributed to cardiovascular diseases (CVD) in 2019 alone [1]. At the same time, CVD and associated risk factors are causing substantial morbidity in patients worldwide [2]. Current treatments for CVD include medications, surgery, medical implants, mechanical devices, and rehabilitation [3,4,5]. Moreover, conventional surgical procedures such as grafting bypass, improve vessel patency, and vascular repair treats more severe conditions of CVDs, e.g., stroke and heart attack [6]. Specifically, innovative approaches like vascular tissue engineering (VTE) have been intensively explored to address the pathophysiology underlying CVD progression and improve the overall life quality of CVD patients through the direct replacement of damaged vessels [7,8].

Currently, VTE lies at the intersection of several emerging disciplines including material science, polymers, stem cell biology, and fabrication technologies to support the development of micro/macroscopic artificial and bioartificial vessels [9,10,11,12]. Different classes of vascular tissue equivalents have been successfully developed as potential replacements for damaged or malfunctioning blood vessels through advancements in human cell biology and cardiovascular physiology [13]. To engineer such artificial blood vessels for in vivo transplantation in patients or in vitro models of vascular pathophysiology, appropriate polymeric materials, cell culture technology, controlled microenvironment, and additive manufacturing are required to develop vascular scaffolds with varied complexity [11,13,14,15,16,17].

Several natural and synthetic polymers have been applied to fabricate biodegradable and biocompatible vascular scaffolds through combinations of chemical processing and manufacturing technologies such as hydrogelation [18], 3D bio-printing, electrospinning, casting/molding, laser degradation, phase inversion, sheet-based fabrication, medical textile (braiding/weaving/knitting), and gas foaming [19,20,21,22,23,24]. For instance, vascular scaffolds composed of poly(ε-caprolactone) (PCL) and collagen fabricated by electrospinning has shown higher durability than sole-PCL/poly (lactide-*co*-glycolide (PLGA) scaffolds [25]. The PCL/collagen composite scaffolds could bear long-term high pressure caused by loaded-volume blood flow and provide a favorable environment for vascular cell growth [26]. 

Scientists have also used poly-L-lactic acid (PLLA)/PCL added with heparin to create small-scale vessel substitutes through electrospinning and extrusion [27,28]. Apart from these examples, other polymers such as PU [29], gelatin [30], chitosan [31], PVA [32], PEG [33,34], PLCL [35,36,37], PGA [38], and PET [39] have been utilized to create multi-scale blood vessel replacements. The general strategy combines two or more of those polymers and fabricates them via the techniques above [14]. Moreover, these polymer-based tissue-engineered vascular grafts (TEVG) have enhanced biomechanical properties, which can better withstand in vivo blood pressures and establish sustainable cellular environments over long periods. Nevertheless, TEVGs must mechanically match the region of interest for transplantation purposes to provide the necessary degree of structural integrity, biocompatibility, biodegradability, and physiology functions [21]. Based on the numerous conditions that must be satisfied, research is still required to optimize TEVG technologies.

This review provides holistic perspectives on recent advances in polymer sciences and fabrication technologies employed to engineer multiscale acellular vascular segments. Secondly, the characterization of the physical and mechanical properties of current TEVGs fabricated from various polymers and technologies is presented and discussed. The biocompatibility, biodegradability, and neovascularization of polymeric vascular scaffolds pertaining to the development of multiscale vascular substitutes are also evaluated. Lastly, significant challenges and obstacles impeding the clinical translation of advanced tissue engineering approaches utilized in TEVGs are identified, along with the exploitation of latent solutions and future directions.

## 2. Methods for TEVGs

As we mentioned in the introduction, the current strategy for creating TEVGs is based on incorporating various polymers with multiple engineering/fabricating technologies. Interestingly, different combinations of polymers devised using similar technologies will present distinct features [11]. Likewise, combining the same polymers with different fabrication methods can create vascular analogs with significantly different physiological and mechanical properties [31,32]. Moreover, the addition of growth factors, peptides, and other physical/chemical cues to the polymers will also impact the performance and function of the resulting scaffolds [40,41]. These attributes highlight the complexity and diversity of TEVG-based approaches. Therefore, identifying various polymers and their corresponding engineering techniques can support vessel replacements with desired features and aid future advancements.

### 2.1. Hydrogelation of Polymers

Blood vessels, mainly categorized as arteries, veins, and capillaries, nourish tissues and organs to support hemostasis and physiological functions. Generally, blood vessels with an inner diameter (ID) greater than 6 mm, between 1 and 6 mm, and less than 1 mm are defined as macrovessels, small vessels, and microvessels, respectively [42]. These three main categories vary in cell composition, histological organization, and physiological functions. However, all three types of vessels possess an innermost endothelial cell (EC) layer while having different compositions in their conduit walls [43]. For instance, as the size of the vessel increase, so does its structural complexity to accommodate more intricate extracellular matrix (ECM) components like smooth muscle cells (SMCs) and myofibroblasts and their secretions [44]. To engineer implantable and bioactive TEVGs, the directed assembly of ECM constituents, such as biopolymers and ECs, onto endothelium equivalents plays essential roles. These components dominate in vivo vascular development by maintaining structure and regulating physiological processes like blood flow [45]. Therefore, to recreate a microenvironment to support these processes in TEVG, biomaterial scaffolds must be designed to enable cell integration, differentiation, and morphogenesis at the transplantation sites. 

The polymeric hydrogel has become a valuable 3D scaffolding unit for fabricating vascular segments as it possesses high water content, good viscoelasticity, biocompatibility, biodegradability, and bioactivity comparable to the natural ECM [46]. These features in the hydrogel stem from the highly cross-linked network of hydrophilic polymers, which can be natural, synthetic, or semi-synthetic (hybrid) in origin [47]. These polymers’ physiochemical properties, initial concentrations, and molecular conformation are engineered to create hydrogel with highly defined properties like pore size, thickness, and durability. Moreover, natural protein-based hydrogels composed of fibrin or collagen enable the derivation of homogenously distributed capillary-based structures ex vivo [48,49]. These compounds also provide mesoscopic templates for seeding ECs and the relevant stem cells to promote further capillary development [50]. Specifically, collagen type I, a native component of ECM, has the intrinsic ability to self-assemble within hydrogels [51]. This property can trigger micro-capillary formation [49]. In addition, collagen-derived gelatins can also form associative polymeric hydrogels with tunable mechanical properties, adding another dimension to retain the complex traits found in various vascular segments. 

Similarly, natural polysaccharide-based hydrogels such as hyaluronic acid (HA), sodium alginate, and chitosan have been intensely evaluated for VTE since they also have physiochemical properties similar to the natural ECM [52]. These properties arise mainly from glycosaminoglycan, peptide, and glycolipid components. Such natural biopolymers have been proposed for localized treatments of cartilaginous lesions as they do not trigger significant inflammatory responses [53]. Moreover, the thixotropic nature of natural polysaccharides allows them to be easily extruded and fabricated into various formats, offering new applications in minimally invasive surgeries [54]. However, traditional hydrogel-based vascular segments do not provide the required mechanical properties [55]. Therefore, various modifications are applied to improve the hydrogelation-based derivatives in natural biopolymers to better mimic the mechanical and physiological features of native vascular scaffolds [56]. For instance, photo-cross-linkable hydrogels can be created by incorporating methacrylic anhydride in the gelatin matrix. The photo-crosslinking of gelatin, as mentioned above, produces a mechanically rigid scaffold that can be used in capillary networks [57]. Moreover, scientists have been trying hard to chemically modify these polysaccharides to improve their suitability for load-bearing structures [58]. 

In contrast, synthetic polymeric hydrogels can be easily engineered with proper mechanical strengths for vascular graft fabrications [59]. For instance, polyethylene glycol (PEG) and related polymers, which are petrochemical products, have an established history of biomaterial scaffold development in vivo. PEG’s application potentials for tissue engineering have stemmed from their low cytotoxicity, stable mechanical properties, and high water affinity [60]. Another class of promising synthetic polymers are poly (lactic-co-glycolic acid) PLGA and polyglycerol sebacate (PGS) gels. The two polymers have been used to fabricate complex microchannels seeded with ECs, which were applied with limited success due to their heterogeneous physiological behavior among cell repopulation [61]. Nowadays, newer synthetic polymers are inclined to be modified from common precursors to improve the biomaterial scaffold’s physiochemical properties. A typical example is a PEG-derived scaffold template known as polyethylene glycol diacrylate (PEGDA). This template is synthesized by acylating PEG and has been shown to promote endothelial tube formation in vitro [62].

#### 2.1.1. 3D Printing 

**Figure 1:**
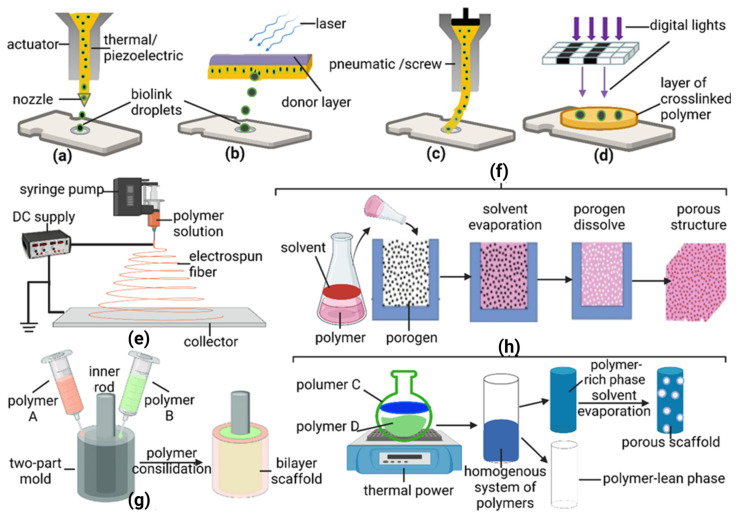
The general process of technologies applied on TEVGs. (**a**) 3D printing-injection, (**b**) 3D printing-laser assisted, (**c**) 3D printing-extrusion, (**d**) 3D printing-stereolithography, (**e**) electrospinning, (**f**) casting, (**g**) molding, and (**h**) thermal-induced phase separation (TIPS) of phase inversion.

In recent years, 3D printing has been used to create cellular and acellular vascular tissue templates [63]. In this section, acellular or polymeric vascular scaffolds generated using 3D printing will be discussed in detail. 3D printing systems are based on computer-aided design (CAD) in combination with layer-by-layer fabrication processes [64]. Inkjet, micro-extrusion, and laser-assisted printing, which can be integrated with 3D printing systems, have emerged as three effective dispersion techniques for fabricating 3D vascular analogs (Figure 1a–h). 

**Figure 2:**
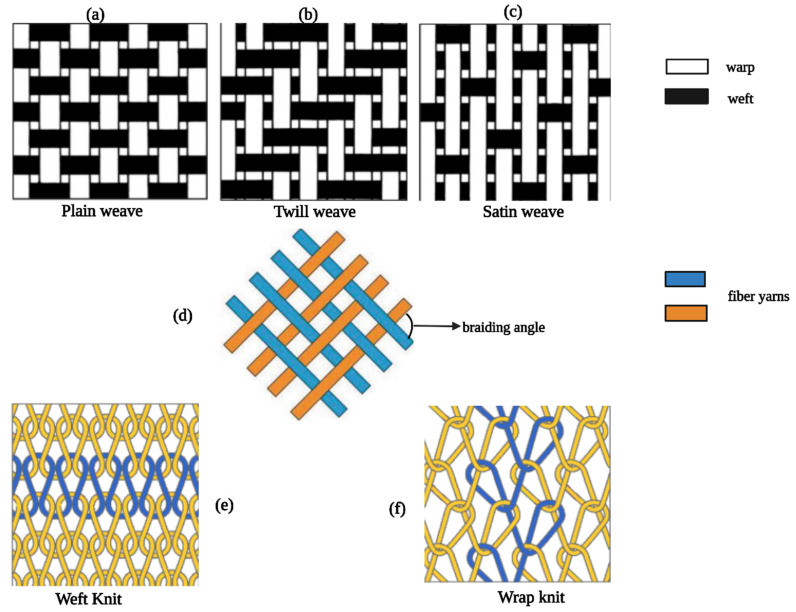
Design patterns of weaving/braiding/knitting. (**a**–**c**) are three types of weaving designs, (**d**) regular structure of braiding, and (**e**,**f**) are two types of knitting designs.

Extrusion-based printing is generally compatible with colossal polymers [65]. The main types of extruders applicable to 3D printing are pneumatic pistons, mechanical pistons, or screw extruding systems (Figure 2c). On the other hand, inkjet-based printing ejects material droplets from a nozzle through thermal or piezoelectric driving forces (Figure 2a), onto a substrate with a specific requirement for the ink’s viscosity (<15 mPa·s) [66]. The material commonly used for inkjet-printed vascular scaffolds is alginate (a natural polysaccharide) [67]. Moreover, laser-assisted 3D printing systems use light energy to crosslink photosensitive polymer precursors (Figure 2b,d). In this category, stereolithography [68] and two-photon polymerization (2PP), as well as digital light processing (DLP), are commonly employed for the fabrication of polymeric vascular grafts.

Many polymers compatible with 3D printing techniques have been applied to generate small- and micro-scale vessels with different dimensions, morphologies, and geometries [69]. In general, such class of 3D printable polymeric materials includes polycaprolactone (PCL)-, gelatin-methacryloyl (gelMA), alginate-, silicone-, polyurethane (PU)-, poly(propylene fumarate) (PPF)-, polytetrahydrofuran-diacrylates (PTHF-DA)-, poly-l-lactic acid (PLLA)-based scaffolds, and other variants that are functionalized by other natural/synthetic polymers or chemicals [29,70]. For example, acellular bioinks for extrusion are mainly composed of PCL, GelMA-gellan, alginate, and silicone occasionally blended with other supportive components such as poly(vinyl alcohol) (PVA), fibrin, Cacl_2_, alginate, and PDMS. Recently, PCL-PVA (support) branched vascular scaffolds with diameters between 2–4 mm have been successfully fabricated with 3D printing techniques (Table 1 [32]. GelMA-gellan-PCL scaffolds with supplementary amounts of alginate and cellular gelatin, which were seeded with mesenchymal stem cells (MSCs), were fashioned into straight vessels with a diameter of roughly 4 mm [32]. Besides, PPF and PU are typical polymeric scaffolds created through the DLP technique. In this technique, 3D structures were generated from a combination of PPF and PU and illustrated a way to greatly improve manufacturing efficiency [71,72]. Likewise, PTHD-DA-based segments fabricated by 2PP can produce branched microvessels with ID 18 μm. This process provides the highest spatial resolution in 3D-printed products, whereas PTHD-DA-based analogs fabricated by SLA can effectively produce small-scale vessels with ID of 2 mm [73,74]. Additionally, 3D inkjet-based printing systems work well with CaCl_2_ bathed in alginate. This technique produces scaffolds that can support the integration of SMCs, leading to the formation of straight micro-scale vessels with ID of 2 μm [75]. Further details on these approaches can be found in Table 1.

#### 2.1.2. Electrospinning

Electrospinning technology works well with synthetic/natural polymers or a combination of various polymers in solutions for manufacturing TEVGs [76]. Briefly, electrospinning employs high electrostatic force for driving the transformation of the viscoelastic solution into nano/micro-fibers [20]. There are three different schematic setups of electrospinning to create distinct polymeric scaffolds. Generally, a typical electrospinning setup (Figure 2e) involves a rotating mandrel, syringe, and syringe pump for forming multilayered composite tubular scaffolds [20]. Alternatively, only a single rotating mandrel is used in another setup to fabricate two hybrid polymers [77]. The other system, which includes electrospinning and electrospraying, generates different scaffold morphologies such as hydrogel and fiber from the polymer solution [78]. The hydrogel can also be electrosprayed as a prepolymer, incorporating the advantages of a hydrogel and of polymers [79]. 

A broad range of polymers have been used in electrospun tubular scaffolds, including polyurethane PU, polyethylene oxide [80], poly (D, L-lactide-co-glycolide) (PLGA), and poly-caprolactone (PCL). These materials are applied as composites for building vascular grafts [81,82,83,84]. Some of those polymers can be blended with natural polymers to enhance cellular attachment and infiltration, while others are a combination of different synthetic polymers to mimic multilayer-tubular models, similar to native blood vessels (Table 1) [85]. For instance, segmented polyurethane (SPU) blended with styrenated (ST)-gelatin and type I collagen can form multilayered structures [86]. Furthermore, SPU and PEO were simultaneously electrospun to create complex fibers. The multilayering electrospinning of PLGA and the addition of elastin and type I collagen resulted in vascular scaffolds with superior biocompatibility and attractive mechanical properties [87]. Moreover, scientists have been fabricating a vessel graft containing PCL, collagen, PEO, gelatin, and Heprasil, although its mechanical properties were not revealed [88]. 

### 2.2. Casting and Molding 

Apart from 3D printing and electrospinning, casting and molding are traditional technologies used to generate vascular scaffolds [89]. With respect to casting, the polymer is dissolved in a solvent, and then a porogen (pore generator) is put into the solution (Figure 2f). As the evaporation of solvent proceeds, a porous structure is created through a leachable porogen [90]. Porogen size and concentration can control the pore size and porosity of the scaffold. Fabrication by casting is relatively easy, but the resulting mechanical properties of the scaffold are inferior compared to those generated from other methods [91]. Therefore, a lock of vascular replacement is still made solely through casting. Castings are typically combined with electrospinning to create vessel grafts, illustrated in the combined fabrication strategies [90]. 

Some molding procedures are similar to those of casting, but molding can fabricate polymer-based scaffolds with customized shapes, which is achieved via a hollowed-out mold [92]. In general, the mold has the external shape of desired products. Once the casted polymer solution is solidified in the mold, the mold will be removed, and the final scaffold will be produced [93]. By altering the geometry of the molds, the customizations of those vascular replacements with different shapes are achievable [94]. Both tubular-shaped vascular grafts and complexly branched vasculatures can be obtained through molding. Several variants of this technique include injection molding, inserted-mesh molding, electrospun-coating molding, micropatterned-surface molding, and sacrificial molding [95]. In conventional injection molding, the polymer solution is cured inside an annular mold composed of an inner rod to set the inner diameter of grafts and a hollow outer tube to control the wall thickness [85]. Additionally, the injection molding is recommended to incorporate pore-generating procedures such as salt leaching, gas foaming, and phase separation in order to obtain porous molded vascular grafts (MVGs) with improved cell infiltration [96]. Natural biopolymers like collagen, HA, silk, and fibrin and synthetic polymers such as PEGDA, PGS, and Poly(l-lactide-co-ε-caprolactone) (PLCL) are applicable for injection molding for the fabrication of vascular grafts with or without the admixture of cells [97,98,99,100,101,102]. 

Mesh insertions are also employed in the underlying framework during MVG fabrication to enhance the mechanical integrity of layered tubular molds. This type of meshed network can be developed from polymeric materials such as P(L/D)LA, Dacron (polyester), poly(glycolic acid) (PGA), PLLA, and PLGA [102,103,104]. For example, a porous mesh containing melt-spun (P(L/D)LA) fibers was applied to increase the burst pressure of a fibrin-based knitted MVG. Moreover, PGA-, PLLA-, and PLGA-containing meshes have generated porous frameworks of vascular tissue analogs with satisfactory mechanical properties [105]. Apart from this, electrospun fibrous films have been coated on MVGs to prevent blood vessel rupture, another method built upon molding [85]. Alternatively, PEUU, PU, and PLA are frequently employed as electrospun coatings outside vascular grafts to strengthen withstanding pressure [106]. For instance, (PLA) nanofiber coated on porous PLCL tubes assures the vascular grafts of bearing the pulsatile pressure in the murine aorta [107,108]. 

ECs and SMCs play essential roles in blood vessels’ tubular formation and maturation [95]. The development of MVGs via micropatterning the inner/outer surfaces of vascular replacements is to change the scaffolds’ surface topography. Specifically, the surface topography of tissue-engineered vascular grafts (TEVGs) has been shown to influence ECs’ adhesion and proliferation along with the recruitment and alignment of SMCs [83,109]. Therefore, engineering the surface topography of MVGs through micropatterning polymers to promote vasculogenesis is an alternative for vascular graft fabrication with high potential. Moreover, PDMS and a proportional mixture of ε-caprolactone, l-lactide, and glycolide have been demonstrated in their applications as TEVGs [110]. For example, a thin layer of PDMS was patterned onto the surface of an inner rod of an annular mold before casting with another polymer solution, which formed a perforated microfluidic device to sprout angiogenesis [111]. Another study illustrated that the micropatterning of the external surface of MVGs with a mixture of ε-caprolactone, l-lactide, and glycolide facilitated the circumferential alignment of SMCs and augmented the expression of SMC’s contractile proteins, which cannot be observed in non-micropatterned MVGs [112].

Sacrificial molding is a technique that employs sacrificial polymers, creating mesochannel-like patterns to be embedded within a synthetic ECM. Subsequently, the polymers will be removed (this is why they are called “sacrificial polymers”), leaving synthetic ECM-embedded mesochannels for cell seeding and nutrient perfusion [94]. For instance, PDMS and PVA were used in fabricating mesochannels in molded scaffolds composed of collagen, fibrin, and Matrigel or in porous hydrogel scaffolds consisting of HEMA and GelMA. Eventually, PDMS and PVA were removed by physical removal and water dissolution [113]. Afterward, synthetic matrices were ready for EC sprouting and remolding for vasculature fabrication [114].

### 2.3. Laser Degradation

Laser degradation is another method to generate blood vessel replacements, wherein a high-energy laser has been used to specifically degrade cross-linked hydrogels (cellular or acellular) for creating patterned constructs [85]. A wide range of natural/synthetic polymers (elastin, collagen, silk, agarose, PEG, PEGDA, PMMA) containing high contents of ECM proteins has been applied in laser degradation processes. Those polymers are used as hydrogels in most situations based on their two-photon absorption profile [115,116,117]. The mechanism uses the laser to induce low two-photon absorption in polymers (PEG/PMMA) that will generate free electrons for physical degradation. At the same time, acute heat accumulation and chemical protein denaturation induced by high two-photon absorption by natural polymer composites containing collagen, silk, and polymer/protein further facilitate hydrogel degradation [118]. Thus, laser degradation is an appropriate technology for establishing microvasculature. In a study, 3D cerebral capillary networks were created through laser degradation exerting on PEGDA hydrogels where the elongation of microchannels was presented. The capillary constructs displayed high fidelity and good alignment [116]. 

### 2.4. Phase Inversion (PI)

Another technique, phase inversion (PI), is instrumental for converting polymer solutions into 3D networks and is applied in many scenarios for blood vessel analogs [119]. There are four categories of PI based on different parameters controlling the process, namely non-solvent-induced phase separation (NIPS), thermal-induced phase separation (TIPS), vapor-induced phase separation (VIPS), and evaporation-induced phase separation (EIPS) [120]. Regarding the techniques used for generating small-ID vascular scaffolds, TIPS is a practical approach in several studies [121]. In this technique, a homogeneous system with multi-components composed of polymer, solvent, and other solutes will be separated into polymer-rich and polymer-lean phases under predetermined conditions. The system becomes thermodynamically unstable under certain specifically engineered circumstances [122]. The polymer-rich phase will be converted into a porous scaffold following solvent removal. In detail, the solvent can be eliminated through extraction, evaporation, or sublimation, a crucial procedure for obtaining polymeric foams with high porosity [123]. Polymers like PU, PLGA, PLA, collagen, PCL, and poly(3-hydroxybutyrate-co-3-hydroxyvalerate) (PHBV) have already been applied in PI technology with or without the inclusion of other inorganic compounds like calcium silicate (CaSi) or natural polymers such as chitosan, gelatin, or synthetic polymers [124]. For example, PCL-based scaffolds with high porosity and pore interconnectivity have been created through TIPS, as demonstrated by Kozehkonan et al. by coating the scaffold with chitosan (CS), bioactive glass (BG), and gelatin nanoparticles (GEL) [105]. TIPS is often used for scaffold fabrication with other techniques, such as electrospinning [124]. Details about this combination will be discussed in the following sections. 

### 2.5. Sheet-Based Fabrication 

In addition to the technologies mentioned above, sheet-based engineering with cell or polymer layers to generate functional tissues is also widely applicable in the generation of blood vessel prostheses [125,126]. Specifically, polymeric sheets are an intermediary tool for sandwiching cell sheets, as these polymer sheets support cell attachment and proliferation [35]. Polymer sheets are created via electrospinning and molding, whereby cells are cultured on top of those polymer sheets [35]. Besides, the surface patterning of polymeric sheets to enhance cellular alignment and recruitment can be achieved through microcontact printing or related techniques. Moreover, PLLA, PCL, Poly(N-isopropylacrylamide) (PNIPAM), and PDMS have been fabricated into sheets by molding or electrospinning for the seeding of fibroblasts (FBs), ECs, SMCs, and MSCs, as well as the derived cell sheets [35,36,37]. For instance, cells cultured on PNIPAM sheets can form a confluent monolayer at a temperature of 37 °C due to the high hydrophobicity of the thermal-responsive PNIPAM above the lower critical solution temperature (LCST) [127]. Once the temperature falls below the LCST, e.g., 32 °C, the PNIPAM becomes more hydrophilic and swells rapidly after substantial water adsorption, resulting in the detachment of the cellular monolayer from the PNIPAM polymer surface [128]. 

### 2.6. Braiding/Weaving/Knitting

Braiding, weaving, and knitting are standard fabric design techniques and have also been applied to generate replacement vascular segments since 1952 [129]. After several clinical trials of 6–20 mm braided/woven/knitted vascular constructions created from polymers like nylon (polyamides), Teflon (tetrafluoroethylene), Dacron (polyethylene terephthalate), and Orlon (polymerized acrylonitrile) were conducted [130,131]. Nowadays, vascular substitutes with diameters between 3–7 mm and 1–2 mm can be generated through textile fabrication techniques [129].

Fabricating TEVGs from braiding techniques involves the interwinding of three or more fiber yarns under specific conditions (Figure 1), following the direction of fabric assembly [131]. The technique is up-and-coming for creating small-diameter vascular grafts (SDVGs) and multilayered scaffolds due to the ease in the controls of both braided structures and bending stiffness within the braided tube [132]. For instance, Yasumoto et al. constructed a vascular graft with a small ID (1.5 mm) via braiding silk fibers onto a cylindrical polymer tube followed by coating with the silk fibroin solution [133]. The scaffolds were implanted into rats to replace the abdominal aorta, resulting in ECs’ and SMCs’ migration and alignment [133]. Additionally, braided PET cardiovascular graft was combined with PLGA nanoparticles, resulting in the composite graft significantly reducing early thrombosis and inflammatory responses, in combination with low cytotoxicity and good biocompatibility [39].

The weaving technique is defined as two sets of interlacing yarn (warp and weft) at a 90-degree orientation to each other [131]. There are three types of weaving pattern designs: namely, plain, twill, and satin (Figure 1). In 2011, Chen et al. designed an innovative bilayer arterial graft using weaving-based techniques [134]. The graft comprised the outer layer of higher elastic modulus polymer like crimped polyester and the inner layer with low modulus polymer like poly-trimethylene terephthalate [134,135]. The resulting elasticity modulus of the graft better matched that of natural blood vessels [134]. Besides, spandex (polyester–polyurethane) and Dacron have been combined to produce woven with enhanced mechanical properties for artery substitutes [136,137]. Knitted vascular grafts consist of a looped filament construction in which a continuous interconnecting chain of yarn loops spirals around the graft’s circumference [138]. 

Knitting is like weaving, while the knitted grafts are more soft, flexible, compliant, and easy to handle [129]. The most common types of knitting designs include weft knit and warp knit (Figure 1) [139]. Generally, knitted vascular grafts are made from Dacron, nylon, and Oron. However, Dacron has achieved better clinical performance than the other materials, as mentioned above [140,141]. This process is similar to braiding and has been applied to generate alloy stents; the knitting technique is often used in manufacturing cardiovascular stents [142,143].

### 2.7. Gas Foaming

Gas foaming is another technique applicable to VTE, which can generate porous structures with the approach of gas expansion, including batch foaming, microcellular injection molding, extrusion foaming, etc. [144]. The associated mechanism involves dissolving the gas (CO_2_ or N_2_) in polymeric materials under high pressure by attaining a supercritical state, making it easy to mix with polymer and promoting polymer pore formation [145]. The gas-foaming method was considered a potential methodology for fabricating microcapillary scaffolds since it has demonstrated the ability to form micro-scale pores in macroporous calcium phosphate cement [146]. Moreover, PCL/PLA blends were prepared by CO_2_ gas foaming, resulting in an interconnected porous structure for building SDBV grafts. The grafts were successfully recellularized with HUVECs, eventually exhibiting high viability and migration [147]. 

In other studies, Yu et al. developed polydimethylsiloxane (PDMS) gel-based microfluidic chips coated with the ionic complementary peptide EAR16-II to enhance the water permeability of PDMS gels for enabling cell culture [148]. Semi-synthetic polymers, such as gelatin methacryloyl (GelMA) gels, are applied to produce microcapillary anastomoses in murines via in vitro pre-vascularization or subcutaneous injections [149]. PEG-based scaffolds have been cross-linked with other polymers, growth factors, and peptides to produce semi-synthetic polymers as vascular templates [150]. For instance, photo-crosslinked collagen–PEG, photo-crosslinked fibrinogen–PEG, and peptide-modified PEG gels can generate microcapillary networks and promote cell growth [151]. Specifically, hybrid hydrogels built by the crosslinking of GAG heparin and PEG enable microcapillary networks’ hosting through biological modifications [41]. Scaffolds produced from such covalent and non-covalent conjugation schemes possess sufficient compressive resistance [152]. These scaffolds can also mediate the release of heparin and support the localized presentations of multiple soluble signaling molecules to potentially prevent stenosis formation in artificial blood vessels and support the formation of conduits ranging from single-layered vascular structures to more advanced multi-layered segments. 

In addition, bioactive elements such as growth factors are largely built into synthetic scaffolds for controlled release applications [60,153,154]. Common growth factors include vascular endothelial growth factors (VEGF), fibroblast growth factor (FGF)-2, or platelet-derived growth factors (PDGF). VEGF, FGF-2, and PDGF have been shown to be essential biological supplies for endothelial, perivascular, and stromal cell development and blood vessel maturation [42]. For example, FGF-2 is a potent mitogen for vascular ECs and fibroblasts and an inducer of angiogenesis. Additionally, FGF-2 enhances the physiological functions within the vasculature, such as cell proliferation/migration, proteinase production, and integrins expression [155]. VEGF, another endothelial mitogen, promotes vasculogenesis and angiogenesis by increasing the permeability and fenestrations within capillaries/venules and by regulating vessel differentiation [156]. Furthermore, members of the PDGF family, including PDGF-B and PDGFR-β, can augment vascularity and accelerate the maturation of vascular vessel walls [157]. 

Interestingly, VEGF and FGF-2 synergistically improve angiogenesis [158]. Some scaffolds that are PEG- and fibrin-based [159] have been decorated with these endogenous compounds that directly promote vascularization within their material matrix [160]. Other modified hydrogel-based scaffolds such as PEG, dextran (polysaccharide), and chitosan [161] have also presented favorable molecular cues for vascular regeneration as admixtures with vasculogenic factors, like arginine–glycine–aspartic acid (RGD) adhesion peptide sequences, and matrix metalloproteinases (MMP) in order to promote cellular integration and scaffold remodeling [162,163]. For example, gelatin-, alginate-, PEG-, and HA-based hydrogels incorporated with vasculogenic factors can successfully induce microvascular growth after implantation in animal models [164,165,166]. Moreover, RGD-modified scaffolds can bind variable types of vascular cells like ECs and smooth muscle cells (SMCs) to create more complex vascular components in vitro, demonstrating an ability to anastomose with other host vessels in vivo post-transplantation [167]. 

### 2.8. Combined Techniques 

The development of bioartificial/artificial vasculature is highly complicated since the requirements of successful vascular grafts include nearly perfect bio-mimeticity and adequate mechanical strength to support the normal physiological function of blood vessels [168]. As discussed above, several emerging technologies have both pros and cons, strongly implying that there are many techniques for fabricating ideal vessel grafts. Combinational techniques emerge as a preferable and promising strategy to generate multiscale, functional, and durable vascular analogs [169]. 

Electrospinning, 3D printing, casting/molding, hydrogelation, and other complementary techniques are usually integrated to develop blood vessel replacements [19]. For instance, 3D printing has emerged as the primary contributor to the fabrication of complex geometrical structures, when the support material would be removed during fabrication [170]. Combining casting/molding, hydrogelation, and 3D printing will eliminate these sacrificial materials via mold formation in the casting/molding process, and the desired constructs can be produced massively [171]. For instance, 3D extrusion printing and casting were combined to fabricate vessel scaffolds wherein Carbopol (a polymer) was used as a sacrificial polymeric gel. Since the Carbopol gel is fragile, it was extruded by a 3D printer and then overlain by the material, which was eventually framed by casting. After that, the casting material was cured, and the gel was removed [172].

Besides, Nie et al. also demonstrated that an integrated strategy involving mold casting, 3D printing, and hydrogelation to create vascular replacements was highly effective. Ultrafine fiber networks established that blood vessel channels were created through 3D printing. Gelatin was cross-linked twice, cast into fiber networks, then formed into a hydrogel sheet. When hydrogels are cured at 4 °C under UV light, fibers are removed to obtain scaffolds with internal vasculature [19,173]. Both strategies as mentioned above were applied to generate multiscale and individualized vascular analogs [174]. 

Recently, 3D printing and electrospinning have been combined to generate novel vascular grafts with small IDs and pore sizes, as well as high porosities [175]. For instance, vascular constructs that are uniaxially micropatterned were fabricated by using extrusion 3D printing of PCL and collagen. Continuously, these constructs were coated by polyethylene oxide/alginate, a form of fibers from electrospinning, followed by the seeding of endothelial cells [19,176]. Another study by Lee et al. showed that UV-assisted 3D printing could be used to prepare a polyethylene (glycol) scaffold embedded with electrospun PCL/gelatin microfibers to improve vascularization. 

Additionally, there is a new integrative combination of various complementary approaches which involve Lego-like construction, e.g., 3D printing and casting. Lego-like construction is a process for assembling individual scaffolds into complex vascular-construct networks [19]. It is because structured blood vessel scaffolds fabricated from a single technique fail to function under most physiological scenarios. These individual scaffolds with diverse functions in the blood vessel can be assembled into complex geometry by 3D printing [177]. The assembly of essential components into a comprehensive vascular scaffold is the development of connectors, especially cylindrical connectors between scaffold modules, which will be produced by 3D printing and casting together [178]. For example, scientists designed a miniaturized hollow microcage as the basic module for vascular graft via the DLP 3D printing technique and assembled these basic modules to form an intact vessel tube. The porous structure of basic modules enhanced cell invasion and vascularization [179]. Specifically, polymer PDME is applicable in this combinational technology. In a recent study, Zhu et al. developed vascular scaffolds with microchannel networks by casting, while a basic scaffold model was fabricated by stereolithography. Subsequently, metal particles were sprayed on the primary mold. Subsequently, PDMS was poured into the mold, leading to the basic PDMS modules being obtained by demolding. Finally, those components (modules) were connected and assembled to a complex vascular vessel scaffold [180]. 

In addition, electrospinning can be combined with PI to generate small ID vascular grafts. For example, a group has recently fabricated poly(ester urethane) urea scaffolds by TIPS, followed by the outer layer deposition of the same polymer as a reinforcement of TIPS scaffolds by electrospinning [181]. Then the compound scaffolds were completely seeded with allogeneic, LacZ-transfected, muscle-derived stem cells (MDSCs), creating new vascular constructs that were implanted as the abdominal aorta of rats for 8 weeks [78]. In an exception, Wu et al. fabricated a cell-free synthetic vessel graft through the utilization of similar approach involving both TIPS and electrospinning [182]. This vessel graft contained two layers, with the outer layer of the PCL sheath fabricated by electrospinning and a core layer with poly(glycerol sebacate) (PGS) developed by a salt fusion and leaching method. A mandrel and a 1.25 mm outer mold were applied to form the outer layer, and a PCL solution was directly electrospun onto the rotating mold [183,184]. A synthetic scaffold was created following the removal of the salt. Three months post-transplantation in rats, as a replacement of an abdominal aorta, it was shown that most parts of the graft resembled native arteries [183]. 

## 3. Design Considerations

### 3.1. Properties of Native Blood Vessels

Blood vessels are mainly categorized as arteries, veins, and capillaries, which nourish cells, tissues, and organs, leading to the substantiation of homeostasis [185]. In other words, normal blood vessels stabilize blood pressure and promote nutrient exchange and waste elimination. Generally, blood vessels can be characterized by their calibers, i.e., inner diameter (ID), under three main classes, as follows: macrovessels (ID > 6 mm), small vessels (1 < ID < 6 mm), and microvessels (ID < 1 mm). Interestingly, vessels under these three classes are different in composition, histological organization, physiological function, and mechanical properties [46].

Arteries/veins comprise three tissue layers, known as tunica adventitia (tunica externa), tunica media, and tunica intima, while capillaries are made of a single endothelial layer with a support basement membrane [129,186]. Moreover, there are three types of capillaries, including continuous, fenestrated, and sinusoid (discontinuous) structures, defined explicitly by their porosity. In arteries/veins with macro/small/micro inner diameters, the vessel wall thickness and molecular composition of the structural components in each vascular layer (collagen, elastin, endothelial cells, smooth muscle cells, etc.) are substantially different from others. Those differences have resulted from the variation in characteristic phenotypes against the change in blood pressure, porosity, permeability, and elasticity. The blood pressure of arteries (90–100 mmHg) is significantly higher than that of veins (5–15 mmHg) [187]. Besides, large arteries have a higher content of elastin to collagen (~1.5) for the promotion of stretching and recoiling during systole and diastole, while medium and small vessels contain fewer elastic fibers since they are required to perform a relatively low degree of stretching [188]. The wall thickness of the vein is significantly thinner than that of arteries, especially within the tunica media. Moreover, the proportion of elastin to collagen in veins is smaller (~0.3) than that of arteries [158]. 

The physiological functions and blood pressure requires the attainment of a specific range of mechanical parameters of diverse blood vessels, such as elastic modulus [189], ultimate stress [189], strain at failure (%), and burst strength (mmHg) [185]. For instance, in the longitudinal and circumferential direction, the saphenous vein (small ID)’s elastic modulus can be 130 and 4.2 MPa, respectively, or 23.7 and 2.25 MPa, respectively. Their burst strengths under circumferential elastic modulus are between 1680 and 3900 mmHg when the elastic modulus is 4.2 MPa and 1250 mmHg when the elastic modulus is 2.25 MPa. [107]. The left internal mammary artery (small ID)’s elastic modulus along longitudinal and circumferential directions are 16.8 and 8 MPa, respectively, with corresponding ultimate stresses determined as 4.3 and 4.1 MPa. Moreover, its circumferential burst strength is 2000 mmHg [190,191]. The major femoral artery, whose ID is 7.02 ± 0.85 mm, attains an elastic modulus of 9–12 MPa along the circumferential direction [192,193]. A more detailed description is shown in Table 2. 

With respect to porosity and permeability, blood capillaries at the micro-scale have been primarily discussed. Only now, the exact value of pore size and permeability of capillaries has been reported [194]. However, the term “vascular permeability’ indicates that the basal vascular sieving of solutes and small molecules occurs under unstimulated circumstances [195]. The extravasation of molecules more prominent than 40 kDa requires the proactive disruption of the vascular barrier, which mostly happens at the post-capillary venules [196].

### 3.2. Major Requirements for Effective TEVGs 

#### 3.2.1. Porosity and Permeability 

As aforementioned, intact and functional natural blood vessels incorporated with high porosity and permeability are also essential for driving the physiological functions of TEVGs [185]. In general, the porosity of TEVGs can be controlled and adjusted by changing the parameters of fabrication techniques like electrospinning [185,197]. Engineering porosity in the vascular graft is not only related to nutrients and waste exchange but also affects the mechanical strength of vascular grafts [198]. Furthermore, permeability directly dictates the effectiveness of molecular exchange between vascular replacements and the surrounding microenvironment [185]. Thus, an artificial vascular conduit with a precisely engineered permeability may be capable of achieving molecular exchange while limiting the passage of immunogenic molecules to enter vessel analogs [199,200]. 

#### 3.2.2. Mechanical Properties

There are three major mechanical properties of TEVGS that must be addressed: mechanical strength, elasticity, and compliance [185]. TEVGs should comply with the existing autologous blood vascular network post-transplantation and provide sufficient mechanical strength, as well as elasticity to withstand blood pressure [201] and cellular host-cell infiltration [202]. The burst strength of TEVGs is supposed to be at least 260 KPa [185,203]. The elasticity of TEVGs is considered ideal when it approaches the known performance parameters of some natural vessels listed in Table 1. Generally, a thicker and reinforced fibrous graft is preferable [185]. In the human body, cells within tissues are usually found within a distance of 100–200 μm from the adjacent capillary for obtaining oxygen and nutrients [204]. Engineered capillary networks thicker than 200 μm will limit adequate nutrition and oxygen exchange [204,205].

Nonetheless, with an increase in vascular wall thickness, the compliance of TEVGs will be reduced as well, which will adversely affect neovascularization, leading to the failure of TEVGs [185,206]. This is because the compliance of TEVGs refers to its expanding ability during the systolic–diastolic cycle or capacity to absorb cardiac ejection as the connection between luminal pressure and vascular wall deformation [206,207]. Apart from that, compliance is regarded as a key factor influencing the long-term performance of vascular substitutes, which would result in anastomotic-derived false aneurysms and the onset of intimal hyperplasia and stenosis after transplantation [185,208,209]. 

#### 3.2.3. Blood Compatibility 

Once the TEVGs are implanted in vivo, blood will be set to flow through those grafts’ inner linings. Blood compatibility corresponds to the endurance of blood to foreign materials, e.g., vascular substitutes [210]. Moreover, TEVGs might cause thrombosis due to its direct contact with blood, triggered by a cascade of biomolecular events [211]. Firstly, the luminal surface of TEVGs will be deposited with a layer of protein such as Factor XII. Afterward, the protein layer firmly bound on the inner lining of TEVGs induces the aggregation of platelets. Finally, the coagulation pathway is progressed through platelet accumulation and thrombosis formation, leading to the impairment of normal blood flow [212,213,214,215]. Therefore, the anti-thrombosis ability of TEVGs is another pivotal property for the development of engineering vascular grafts. 

#### 3.2.4. Neovascularization/Endothelium Friendliness

Generally, TEVGS will be re-seeded with ECs before or after graft transplantation. ECs adherence to TEVGS is responsible for ECM secretion, preventing thrombosis formation, and assisting in new vessel regeneration [185,216]. In this case, the capability of promoting adhesion and proliferation of ECs for TEVGS implementation should be concentrated on new device evaluation [217]. In particular, engineered vascular replacements’ design and development must address the grafts’ surface chemistry via bioactive molecule coupling or other surface functionalization [11,218,219]. 

#### 3.2.5. Biocompatibility and Biodegradability

Based on the International Standard ISO 10993 (Biological Evaluation of Medical Devices), all materials to be implanted in the human body should be supported by extensive in vitro/vivo tests of biocompatibility to verify the response and behavior of vascular cells interacting with graft surfaces [220,221,222]. A vascular analogy with ideal biocompatibility would support the corresponding cellular activities, including their roles in molecular and mechanical signaling for the promotion of neovascularization, without eliciting any pathophysiological responses from the host [76,223,224,225]. 

Biodegradability is the ability to decompose foreign material into small molecules, which will be transported away from the implant site after attacks from enzymatic/hydrolytic reactions [185,226,227]. Such a process avoids the surgical removal of medical implants caused by a bacterial infection in the long term. The degradation rate of the biomaterial scaffold will influence the inflammatory response by matching with the tissue regeneration rate [228]. The vascular scaffold is required to provide adequate mechanical support during degradation [228]. The biodegradation of polymers is dictated by the hydrodynamic radius (polymer architecture), surface-area-to-volume ratio, degree of crystallinity, physiological environment, and solute composition [229]. Generally, synthetic polymers take a longer time to degrade than natural polymers. For instance, star-shaped polymers and dendrimers with the same molar weight but different hydrodynamic radii demonstrated distinct degradation times. In detail, the degradation time of dendrimers is slower than that of star-shaped polymers [230]. Moreover, polymers in mesoscopic scaffolds or nanoparticles have different degradative behaviors (in vitro and in vivo) due to significantly different surface-area-to-volume ratios [231,232].

### 3.3. Physical and Biomechanical Characterization of Polymeric TEVGs Based on Major Requirements

The overall goal of TEVGs is to act as a biomimetic prosthesis that can maintain homeostasis without any adverse effects (e.g., thrombosis, aneurysm, inflammation) [38,230]. Thus, TEVGs need to fulfill the major requirements of natural blood vessels in vivo. However, the standards for the physical and biomechanical characteristics of blood vessels vary regarding the requirements in various applications [95]. Different materials and technologies used in the fabrication of TEVGs contribute to sets of physical and biomechanical properties in the resulting devices. The subsequent parts of this review will thoroughly discuss the reported physical and biomechanical characterization of typical polymeric (natural/synthetic) TEVGs.

#### 3.3.1. Quantification of Physical and Biomechanical Properties 

##### Physical Properties 

According to the methods prescribed under the industrial standard— ANSI/AAMI/ISO 7198:1998/2001/(R) 2004 (Cardiovascular Implants: Tubular Vascular Prostheses), water permeability is defined and calculated in a scenario wherein permeate steady water flows through vascular tubes for 1 min, followed by collection in a beaker. The water volume collected in 1 min will be measured [233]. The graft permeability (mL/cm^2^/min) was determined by using the following equation:

$$\tau \;=\;\frac{Q}{A}$$

where τ is the graft permeability, Q is the fluid volume passing through the graft, and A is the cross-sectional area of the aperture in the sample holder [233]. 

##### Biomechanical Properties 

Young’s modulus or elastic modulus, referred to as the stress-to-strain ratio when the samples are under deformation, is an intrinsic mechanical property of grafts [234]. This ratio reveals the material stiffness, which is especially crucial for the performance of small ID blood vessels [235]. Both Young’s modulus and fatigue behavior are critical properties of native blood vessels [236] since mismatched stress will expedite the failure of vascular replacement [237]. Human blood vessels generally present a low Young’s modulus of around 20 kPa (commonly 20–50 kPa) circumferentially at the early stage of radius expansion, implying good flexibility. During the later stage of circumferential expansion (30–60% strain), Young’s modulus of the human blood vessels improves sharply, reaching up to 100–200 kPa to constrain its further expansion [238]. These phenomena could be explained as the self-protection of blood vessels from rupture under a sharp and immediate increase of blood flow during exercises or a burst of crying and laughing [239]. 

The burst pressure is the maximum pressure a blood vessel can withstand before an acute leakage and eventual fracture occurs. Burst pressure is expressed in the following equation:

$$f=\frac{Pd}{2t}.$$

where *f*, *P*, *d*, and *t* are the maximum force, burst pressure, diameter of a blood vessel, and wall thickness of blood vessel, respectively [132]. Since *f* should remain constant for a particular type of blood vessel, *P* will decrease when *d* increases or *t* drops. Nevertheless, *P* within a small ID blood vessel can be significantly higher than the systemic blood pressure. For example, the *P* of a carotid artery is close to 5000 mmHg, while its common systolic pressure is just around 130 mmHg [240].

Moreover, the compliance (C) of vascular grafts is determined through the following equation:

$$C=\frac{\frac{R2-R1}{R1}}{P2-P1}\times 104$$

where *R* is the radius of the graft at pressure *P* [233]. The suture retention strength (SRS) measures the adhesion of a graft to surrounding tissues whose resistance to the stresses is exerted by pulling a suture through the blood vessel wall [241].

### 3.4. Physical and Biomechanical Characterization of Natural Polymer-Based TEVGS

Natural polymers are recommended for TEVGs owing to their superior performance on biocompatibility and biodegradability while being readily embedded with porosity and permeability, as well as similar bioactivities of natural ECM for promoting neovascularization after TEVG implantation [242]. Collagen is considered an excellent biomaterial for fabricating TEVGs due to the presence of integrin-binding domains and the negligible induction of an inflammatory response [10]. For instance, collagen type I hydrogels have been used to fabricate perfused microfluidic constructs in vitro, forming 3D microvascular tubes (ID = 116 μm) [18,243]. Then, the microvascular conduits seeded with human vascular cells demonstrated a vital barrier function without causing detectable inflammation [243]. As previously mentioned, the biomechanical properties of the microvascular scaffolds were not revealed, but their respective permeabilities were measured. The permeability coefficient of the bovine serum albumin (BSA) through the luminal walls of the scaffolds was estimated to be 5.5 × 10^−6^ ± 3.5 × 10^−6^ cm/s (*n* = 3) at days 3–4 and 7.9 ± 3.5 × 10^−6^ cm/s at days 6–7 (*n* = 3, *p* > 0.05 by Wilcoxon’s sum of ranks test). Most tubes showed small focal leakage of BSA [243]. 

Gelatin is derived from collagen through its irreversible hydrolyzation, while cell-binding motifs like RGD and degradation sites sensitive to matrix metalloproteinase are maintained. As a result, gelatin has already become a very popular biomaterial for developing TEVGs [244,245,246]. An example of its application is the development of microfluidic vascular scaffolds by crosslinking gelatin with the enzyme transglutaminase showing good cytocompatibility for murine mammary epithelial cells and good nutrient/waste transfer for cells [30]. The physical and biomechanical properties of the gelatin scaffolds were not reported. In addition, fibrin is a biocompatible, elastic protein applied to fabricate vascular substitutes. The good binding affinity of fibrin to critical ECM proteins such as fibronectin, vitronectin, and thrombospondin makes the construction of VEGF through microfluidic devices highly feasible [10]. For example, parallel fibrin hurdles mixed with other hydrogels were generated into a quasi-3D microfluidic vascular model with solid attachment and regrowth/differentiation of adipose-derived stem cells (ADSCs) ) [247].

Polysaccharides (e.g., HA, agarose, alginate, chitosan) with good biocompatibility, biodegradability, and excellent gelation properties are used for fabricating vascular constructs with microchannels [248]. For instance, agarose hydrogels molded into microfluidic channels have efficiently delivered nutrients to encapsulated cells [249]. At the same time, Ling et al. also demonstrated that 3% agarose hydrogel with a reported stiffness of 19–32 kPa is rigid enough to support the 3D architecture of microchannels during the fluid perfusion technique without causing significant deformation [250]. As mentioned above, the main disadvantage of these vascular replacements fabricated from the natural polymer is their poor mechanical properties [251]. Nevertheless, this shortcoming can be improved by adjusting the polymer concentration, crosslinking scheme, and environmental conditions and combining them with synthetic polymers (as mentioned earlier). Therefore, trending research focuses on composite (natural and synthetic) or multi-layered polymers to develop advanced TEVGs, which will be described in the following sections.

### 3.5. Physical and Biomechanical Characterization of Synthetic Polymer-Based TEVGS

A single type of polymer fabricated into vascular substitutes using one manufacturing technology is inadequate for TEVG application [21]. Generally, synthetic polymers are embraced with better physical and biomechanical properties, which are relatively easy to be engineered into particular regimes [206,252]. Meanwhile, the long-term challenges for synthetic-polymer TEVGs stem from the weak surface bioactivity and the difficulty fabricating them into blood vessels with small ID [253,254]. The surface characteristics of TEVGs are indispensable, since those molecules are responsible for triggering subsequent cell adhesion, proliferation, migration, and differentiation for achieving the roles of ECM [254,255,256]. Therefore, in this part, the physical and biomechanical characterizations of synthetic polymer-based composite TEVGs as vascular grafts with small and micro-IDs will be discussed based on the recent findings in the field. 

### 3.6. PEG/PLGA/PDMS/PGS-Based Composite TEVGS

Generally, PEG has a high solubility in water and inorganic solvents (e.g., methanol, ethanol, and dichloromethane) and a low protein affinity, while it is not immunogenic, making it highly applicable in VTE [257,258]. PEG was proved to be non-toxic with good biocompatibility and controlled biodegradability and mechanical properties [259]. At the same time, PDMS is biocompatible, non-toxic, and hydrophobic, while PLGA has demonstrated good biocompatibility and biodegradability without causing cytotoxicity [260,261]. 

In a recent study, PEG hydrogel was incorporated with a collagen-like protein derived from group A Streptococcus, Scl2.28 (Scl2), then reinforced with an electrospun PU to generate bioactive multilayered vascular grafts with small ID (3.7–4.7 mm) [33]. The results showed that the pressure through vascular grafts and the mean peak-to-trough pressure differential were 50 mm Hg and 20 mm Hg, respectively. Furthermore, the circumferential modulus of multilayered constructs was 190 kPa under physiological stresses (100 mm Hg). The lowest suture retention strength of the new graft was determined to be 406 ± 124 gf, exceeding that of other saphenous vein grafts with similar dimensions [34]. Moreover, the maximum burst pressure of vascular constructs with a thickness of 1.19 ± 0.17 mm was measured at 1440 ± 40 mm Hg. The maximum graft compliance was determined to be 5.9 ± 1.4% radial change per mm Hg, which was close to that reported for saphenous vein constructs [262]. Besides, PEG-based vascular scaffolds provided a local microenvironment for supporting rapid reendothelialization and avoiding platelet adhesion, aggregation, and thrombus formation [33]. 

PEG is often chemically coupled with fibrinogen for developing PEGylated hydrogels fabricated into microfluidic constructs [230]. Then the microfluidic channels are repopulated with cells under laser photoablation to promote cell proliferation. The scaffolds have demonstrated good mechanical properties and cell affinity [231]. Moreover, the ratio of PEG to fibrinogen is adjustable, while a higher PEG-to-fibrinogen ratio achieves better cell maintenance than scaffolds with a lower PEG-to-fibrinogen ratio [232]. Furthermore, composite hydrogel containing peptide amphiphiles (PAs) and PEG was developed as an ECM mimetic that acts as a tunable graft through the facile fabrication method. The final grafts, as mentioned above, have porosity reaching up to 35 nm and others with smaller pores of less than 5 nm [263]. The mechanical strength of grafts was described as soft, medium, and stiff, with ranges using the following criteria: 0.1–0.3, 1–4, and 6–8 kPa, respectively. Besides, the grafts were verified to be highly biocompatible and supportive for cell adhesion even at the early stage of cultivation [263].

PDMS and PGS are commonly applied with other materials to form microscale vascular conduits or micro channels through processing with microfluidic devices, electrospinning, or other manufacturing technologies [264,265]. For instance, Yu et al. have built a PDMS (non-water permeable, noncytotoxic)-based microfluidic chip coated with complementary ionic peptide EAR16-II to enhance the blood biocompatibility and for reducing protein adhesion. Alternatively, PGS was fabricated via molding as a medium between parenchymal and vascular compartments to form bioartificial micro-vessel scaffolds. It demonstrated the fact that these micro-vessel scaffolds supported the in vitro cultivation of human skeletal muscle cells (hSkMDCs) and human umbilical vein endothelial cells (HUVECs). The substantial biodegradation of the PGS membrane in association with host blood cell infiltration of the micro-vessels occurring within one week of implantation in vivo [265].

PLGA is often incorporated into collagen-/elastin-based vascular grafts created by electrospinning to tune mechanical elasticity and solve the problem of compliance mismatch [149,266,267]. More recently, PLGA and PGS have been combined to fabricate intricate structures with microchannels for seeding endothelial cells [268,269]. The diameter of the final composite vascular grafts under the dehydrated state and hydrated state ranges from 384 ± 22 nm to 1196 ± 79 nm and 446 ± 69 nm to 1735 ± 103 nm, respectively. Furthermore, the pore areas of those grafts range from 1.92 ± 0.23 μm^2^ to 4.74 ± 0.43 μm^2^. As the different content of PLGA was blended (16–45%), the tensile strength and Young’s modulus of vascular constructs ranged from 2–137 kPa and 2–901 kPa, respectively [270]. The vascular grafts have been proven with ideal biocompatibility and support the metabolization and organotypic arrangement of ECs and SMCs [270].

### 3.7. PLLA/PCL/PLCL/PDO-Based Composite TEVGs

There is no consistent value regarding the physical and biomedical properties of vascular replacements, since natural blood vessels vary, with distinct features. The current research has not proposed related standards. Therefore, the values vary in different articles. However, a vascular scaffold should not collapse and is supposed to have the ability to withstand high physiological pressure, as well as have similar mechanical properties as native blood vessels [26]. For instance, the human arteries have known mechanical properties with peak stress > 1.5 ± 0.5 MPa, Young’s modulus > 11 ± 1.5 MPa, and strain at break > 0.7 ± 0.1 mm mm^−1^ [82,186,271]. However, capillaries’ physical and biomechanical properties remain unknown, and that of some veins is listed in Table 2. The following PLLA/PCL/PLCL/PDO-based composite TEVGs illustrated the possibility of reproducing the characteristics of native blood vessels.

Scientists electrospun PLLA with gelatin and formed vascular scaffolds with favorable physical and biological properties [272]. The scaffolds have ID ranging from 2–6 mm and were analyzed using FTIR and degradation tests. The studies implied that the scaffolds are biodegradable, and the degradation rate was decided by the concentration of gelatin contained. The FTIR tests showed the appropriate presence of composite PLLA and gelatin. The studies implied that the scaffolds are biodegradable, and the degradation rate was decided by the concentration of gelatin contained [272]. The cell culture result has proved that the aligned nano-fibers of scaffolds largely supported the SMCs and improved the proliferation of cells. SMCs and HUVECs oriented toward fiber alignment and cell proliferation along elongation pathways were enhanced in these new scaffolds [272]. Similarly, Lee et al. demonstrated that the highest tensile strength and Young’s modulus of collagen/elastin/PLLA mixed electrospun vessel analogs were 0.83 MPa and 2.08 MPa, respectively, which constitute greatly improved mechanical properties compared to collagen-/elastin-only conduits [273]. 

Except for PLLA, PCL admixed with other polymers has been widely used for creating TEVGs with better performance on physical and biomechanical characterization [274]. For example, PCL/collagen combined vascular substitutes were generated with a tensile strength 4.0 MPa and Young’s modulus 2.5 MPa, which were even higher than the native porcine coronary artery (2.5 MPa and 1.0 MPa, respectively) [26]. Similar scaffolds produced by Tillman et al. reported a tensile strength of 0.9 MPa, compared to that of rabbit native aorta with a tensile strength of 1.3 MPa implanted in vivo [103,275]. Apart from that, Tillman’s scaffolds maintained structural integrity under normal hemodynamic conditions [82]. Moreover, a multilayer small-ID vascular scaffold composed of PCL, PEO, and a thick, porous genipin-crosslinked collagen–chitosan (GCC) hydrogel sleeve was created through electrospinning. The multilayer scaffold consists of 100 μm-thick PCL single fibers and 20 μm-thick two-fiber segments (interwoven fibers of PCL and PEO or GCC) [233]. The scaffold is biocompatible and biodegradable, with promising physical and biomechanical characterizations based on the earlier methods. The multilayer vascular replacement resulted in a high compliance (4.5%), desired water permeability (528 mL/cm^2^/min), resilient burst strength (695 mmHg), and suture strength (2.38 N), supporting its transplantability. Moreover, the graft was able to support the seeding and culturing of vascular ECs and SMCs in vitro and replace the abdominal aorta of rabbits showing rapid cell growth and stable flow perfusion under physiological circumstances [233]. The physical and biomedical characterizations of other PCL-based composite TEVGs are listed in Table 2.

**Table 1:** Polymer-Based TEVGs and Characterizations.

Polymers		Applied Technology	Characterization	References
**Natural Polymers**	Collagen type I	Hydrogelation/microfluid;	Strong barrier function after being seeded with human vascular cells; compliance coefficient of BSA: 5.5 × 10^−6^ ± 3.5 × 10^−6^ cm/s (n = 3) at days 3–4 and 7.9 × 10^−6^ ± 3.5 × 10^−6^ cm/s at days 6–7; ID = 116 μm	[18,243]
		Hydrogelation/laser degradation	D = 50 μm	[115,116]
	Gelatin	Hydrolyzation/microfluid	Good fluidic access and cytocompatibility to murine mammary epithelial cells; microscale	[30]
	Silk	Braiding	Implanted as a rodent abdominal aorta with ECs/SMCs migration and alignment observed; ID = 1.5 mm	[133]
	Polysaccharides: HA	Molding/microfluid/hydrogelation	Efficient delivery of nutrients Stiffness: 19–32 kPa; microscale	[249]
	Polysaccharides: alginate/Cacl2 (addition)	Extrusion/injection 3D printing	Stiffness < 500 kPa; short maturation of SMCs; D = 1–3 mm BT; D = 2 μm ST L = 2 μm T = 2 μm	[276]
	Fibrin	3D-quasi microfluid	Strong ADSCs attachment, regrowth, and differentiation; microscale	[247]
**Synthetic Polymers**	PCL/PVA	Extrusion3D printing	Porosity: 61% with strand space 0.7 mm; 74% with strand space 1 mm; D = 2–4 mm BT	[32]
	PCL/chitosan	Electrospinning/extrusion 3D printing	ST	[31]
	PCL/GelMA-gellan/alginate	Extrusion 3D printing	D = 4 mm ST	[32]
	PDMS/fibrin		A tissue ring of SMCs after being seeded with HASMCs; D = 5 mm ST	[277]
	Silicone		Stiffness: 20–244.78 kPa; Support culturing of HUVECs, HA-VSMCs, HDF-n; D = 0.5–2 mm ST	[278]
	PU	DLP 3D printing	EM = 1.1 MPa No cytotoxicity at highest concentration 26 mgL^−1^; ID = 1.5 mm OD = 4 mm ST	[29]
	PPF		P = 0.35 nm for ID = 2.5 mm support cell culturing of HUVECs, hMSCs, HUSMCs; ID = 2.5 or 1 mm t = 0.25 or 0.15 mm ST	[70, 279]
	PTHD-DA	SLA 3D printing	ID = 18 μm T = 3 μm L = 160 μm BT ID = 2 μm T = 2 mm L = 2 mm ST	[280]
		2PP 3D printing		[280]
	Heparin-releasing PLLA/PCL	Electrospinning/extrusion 3D printing	D = 5 mm L = 6 cm ST	[27,28]
	PGS/PCL/salt	Casting/molding	SRS = 0.45 ± 0.031 N, EM = 536 ± 119 kPa UTS = 3790 ± 1450 kPa BP = 2360 ± 673 mmHg C = 11% ± 2.2%, transplanted as rat abdominal aorta with progressive vascular remolding in 3 months; ID = 720 μm T = 290 μm	[40]
	10% (*w*/*v*) P(CL/LA)/PGLA (sealed)	Casting/electrospinning	SRS = 2.16 ± 0.037 N EM = 17.73 ± 3.09 MPa UTS = 2.93 ± 0.26 MPa BP = 1002.17 ± 181.98 mmHg, support HUVECs’ attachment and proliferation; ID = 1.02 ± 0.5 cm T = 0.21 ± 0.02 cm	[281]
	15% (*w*/*v*) P(CL/LA)/PGLA (sealed)		SRS = 3.20 ± 0.577 N EM = 26.90 ± 6.66 MPa UTS = 4.75 ± 0.97 MPa BP = 1321.66 ± 214.67 mmHg support HUVECs’ attachment and proliferation; ID = 1.01 ± 0.08 cm T = 0.19 ± 0.09 cm	
	PLCL (inner layer)/PGA/PLA (outer layer)	Casting/electrospinning	Cell infiltration in scaffold observed, transplanted as infrarenal aortic graft in mice, maintaining 8-month survival; Outer layer ID = 600 μm, inner layer ID = 200 μm T = 3 mm	[38]
	PEGDA	LD	Elongated microchannels and molecule transportation between unconnected microchannels observed; support HUVECs’ seeding; Microcapillary	[115,116]
	PU/gelatin	PI	P = 2 μm PE = 1.2 ± 0.4 mLmin^−1^ UTS = 2700 ± 400 kPa Support hMSCs’ adhesion and growth	[282]
	PLLA/inner MSCs	Sheet-based fabrication	Patency of 100% in 8.6 weeks; vascular remolding observed, SMCs alignment in 60 days; ID = 0.7 mm	[35,36,37]
	PLCL/FB/collagen		4-week transplantation, patency unknown; ID = 4.1 mm	
	PET/PLGA	Braiding	Small ID	[39]
	Polyester/PTT	weaving	EM = 1056 MPa under pressure 200 mmHg	[134]
	Spandex (over 80% PU)/polyester	knitting	Transplanted as dog abdominal aorta; D = 8–10 mm	[129]
	PLA/PCL	CO_2_ gas foaming	Recellularized with HUVECs exhibiting high viability and migration; Small ID	[147]
	PEG/collagen/PU	Electrospinning/hydrogelation	Mean pressure = 50 mmHg, peak to through pressure = 20 mmHg, circumferential modulus = 190 kPa, SRS = 406 ± 124 gf, BP = 1440 ± 40 mmHg, C = 5.9 ± 1.4%, support rapid endothelialization; ID = 3.7–4.7 mm	[283]
	PLGA/collagen/elastin		Stiffness: 2–137 kPa, 2–901 kPa, support ECs, SMCs growth, dry pore area = 1.92 ± 0.23 μm^2^ wet pore area = 4.74 ± 0.43 μm^2^; dry D = 384 ± 22 nm–1196 ± 79 nm, wet D = 446 ± 69 nm–1735 ± 103	[266,267]
	PA/PEG	Hydrogelation/molding	P = 35 nm, stiffness:0.1–0.3 kPa, 1–4 kPa, 6–8 kPa, cell adhesion observed	[58]
	PGS	molding	Supported the seeding of hSkMDCs and HUVECs	[265]
	PDMS/peptides	microfluid	Enhanced blood biocompatibility and cell adhesion	[284]
	PLLA/gelatin	Electrospinning	Supported SMCs and HUVECs alignment and proliferation and improved cell proliferation; ID = 2–6 mm	[272]
	PCL/collagen		UTS = 4.0 MPa, EM = 2.5 MPa	[285]
	PCL/PEO/GCC hydrogel sleeve		C = 4.5%, water permeability = 528 mL/cm^2^/min, BP = 695 mmHg, SRS = 2.38 N, supported the seeding and culturing of vascular ECs and SMCs in vitro, quick cell growth, and stable flow perfusion; Small ID	[233]
	Elastin/PDO		SRS = 375 gf, C = 3.8%, EM = 9.64 MPa	[286]
	collagen/elastin/PLGA/PLCL		Substantial interactions between SMCs; D = 200–800 nm T = 0.5 mm	[266]
	collagen/elastin/PLLA		UTS = 0.83 MPa, EM = 2.08 MPa	[273]

T = thickness; D = diameter; L = length; ID = inner diameter; P = porosity; BT = branched tubes; ST = straight tubes; BP = burst pressure; UTS = ultimate tensile stress; EM = elastic modulus; PE = permeability; SRS = suture retention strength; C = compliance; hSkMDCs = human skeletal muscle cells; hMSCs = human mesenchymal cells; LD = laser degradation; PI = phase inversion; HUVECs = human umbilical vein endothelial cells; HA-VSMCs = human aortic vascular smooth muscle cells, HDF-n = human dermal fibroblasts-neonatal; FB = fibroblast; HUSMCs = human uterine smooth muscle cells.

Vascular constructs composed of elastin and polydioxanone (PDO) with a small ID mimicking native femoral artery were designed by Sell et al. via electrospinning [286]. The resultant grafts with a component ratio of elastin and PDO with 50:50 have the elastic modulus (9.64 MPa) and strain at failure (65%), which are close to native femoral arteries (9–12 MPa and 63–76%, respectively) [286,287]. Except for this, the study indicated that the composite polymeric vessel scaffold enhanced the in vitro bioactivity of the matrix. The scaffold possessed ample suture strength and compliance needed for a viable graft [286]. Furthermore, collagen/elastin/ PLGA and PLCL have been fabricated using electrospinning techniques. The layered vascular constructs consist of fibers with diameters ranging from 200 to 800 nm. The average is 550 ± 120 nm, and its thickness is about 0.5 mm. The scaffolds are biodegradable and demonstrated substantial interactions between SMCs that were analyzed and concluded through cell morphology, adhesion, and proliferation studies [266]. Further descriptions of the physical and biomechanical characterization of common polymer-based TEVGs are listed in Table 2.

**Table 2:** Mechanical Properties of Native Blood Vessels.

Vessel Types and Axial Directions	Elastic Modulus [189]	Ultimate Tensile Strength [189]	Strain at Failure (%)	Burst Pressure (mmHg)	References
**Saphenous vein circumferential**	43/4.2/2.25	3/1.8/4	11/243/180	NA/1680–3900/1250	[107,190]
**Saphenous vein longitudinal**	130/23.7	13/6.3	17/83	NA/NA	[190,191]
**Left internal mammary artery circumferential**	8	4.1	134	2000	[190]
**Left internal mammary artery longitudinal**	16.8	4.3	59	NA	[192]
**Femoral artery circumferential**	9–12	1–2	63–76	NA	[193]

NA = not available.

## 4. Characterization of Synthetic TEVGs in Clinical Use

Polyethylene terephthalate (PET, Dacron), expanded polytetrafluoroethylene (ePTFE), and polyurethane (PU) are the three major TVEGs that are invested in clinical use [288,289,290,291]. Clinically available Dacron grafts are fabricated via either weaving or knitting in an over-and-under pattern, leading to minimal porosity and creep [292]. Dacron is stable and can persist for more than 10 years after implantation without significant deterioration when applied as macro-scale vascular replacements. They have poor clinical performance and cause thrombus, inflammation, and compliance mismatches when used as small-diameter vascular grafts [293,294]. The compliance of current commercial Dacron TEVGs is 2.0 × 10^−2^% mmHg^−1^ with 42% of two-year patency [295]. 

Polytetrafluoroethylene (PTFE) was patented first in 1937 as Teflon. Expanded ePTFE (Gore-Tex) is the material employed on vascular grafts and manufactured using heating, stretching, and extruding processes, creating a microporous scaffold for firm cell adhesion [136,289]. An ePTEE vascular graft is non-woven, with a node–fibril structure, and performs well as aortic replacements having a 5-year primary patency rate of 91% to 95% but a lower patency rate for being analogs of substitutes with small ID [296,297]. The compliance of ePTEE is 1.5 × 10^−2^% mmHg^−1^ with 42% of two-year patency [259]. Specifically, both Dacron and ePTEE can be bonded to heparin [256]. Heparin-bonded ePTFE aortic grafts presented decreased thrombogenicity and enhanced patency rates at 8 weeks [298]. Heparin-bonded Dacron grafts are commercially available in Europe [292]. Significantly, the heparin-bonded Dacron showed promising wide application of SDVGs such as femoropopliteal bypass grafting, with eye-catching patency rates at 1, 2, and 3 years of 70%, 63%, and 55%, respectively [299].

Researchers prefer using PU for microcapillary scaffolds due to their microstructure [300]. Polyurethanes can be divided into fibrillar or foamy structures, and both tend to lack communicating spaces for potential capillary ingrowth [301,302]. In microporous foamy PU with a 15 μm pore size, relatively little capillary ingrowth can be achieved. Whereas once the pore size increased up to 157 μm, capillary sprouting occurred [303,304]. Although PU grafts possess many exciting features, such as EC growth under inferior hemodynamic conditions, excellent healing, subtle surgical handling, and low suture bleeding, sufficient evidence of the spread use of PU vascular grafts as human peripheral bypasses remains in scarcity because of lacking investigations [305].

## 5. Key Challenges Limiting the Translation of Polymer-Based TEVGs

Ideally, bioartificial blood vessels should possess the structural and functional capacities of native structures [59]. Therefore, identifying the conditions that may lead to deviation from these ideal characteristics is vital for reducing the potential of device failure. It is also essential that these structures be rendered with bio-inertness for supporting somatic growth post-transplantation [306]. To this end, pinpointing the key challenges the current polymeric TEVGs face in clinical translation is extremely necessary. 

As we all know, the endothelium is essential in restricting the movement of water, cells, and protein between intravascular and interstitial compartments [243,307]. Based on the characterization demonstrated above (Table 2), TEVGs solely composed of natural polymers have better performance regarding biological aspects [59]. These microscale vascular conduits are free of considerations regarding biocompatibility, degradability, and cytotoxicity. They are highly supportive of cell repopulation and nutrition exchange. Besides, different natural polymers will create vascular substitutes with specific physical performance. For instance, collagen type I exhibited a vital barrier function after cell seeding.

Moreover, the endothelium has to align on the basement membrane, where collagen type I is the essential component and regulator [18,308]. This characteristic explains why vascular replacements consisting of collagen type I have a vital barrier function and indicates the potential for endothelium regeneration [309]. However, the mechanical properties of these natural polymer scaffolds require significant improvement. Going back to collagen type I, the stiffness of collagen type I is 0.1–18 kPa when the concentration is 3–20 mg/mL [310]. Based on the fact that compliance is the inverse of stiffness [311], the compliance of collagen type I is around 10^−2^ cm/s. The compliance of vascular conduits made by collagen type I conducted with microfluid/hydrogelation is close to 10^−6^ cm/s. The compliance of native micro-vessels with the same dimension is unknown, but the compliance of this polymer has been highly reduced when formed into microvascular constructs. 

However, the mechanical properties of polymers are flexible and changeable by distinct ways of fabrication, physical/chemical reactions, and incorporation with other materials [312]. The HA vascular micro-tubes in Table have a stiffness from 19 to 32 kPa, while, when it combines with PVA as a composite hydrogel, the stiffness can be extended to 200 kPa [313]. Other similar examples provide future research directions on amplifying the mechanical properties of natural polymer-based vascular homologs but also bring new challenges of choosing to fabricate techniques, a combination of polymers, and methods of modifications [312]. These problems and confusion can only be solved with arduous academic work. Besides that, the mechanical properties of these natural polymeric vascular substitutes still need to be discovered, which implies a shortage of small- and macro-scale vessel analogs generated by natural polymers. 

For vascular scaffolds created by synthetic polymers, their dimensions become multiple at the micro-scale level, and the small ID vascular structures have been formed through the braiding of PET/PLGA and the casting/electrospinning of PLGA/ P(CL/LA). Except for this, the morphology of blood vessel conduits is not limited only by straight but also by branched tubes [312]. Most scaffolds’ mechanical features are available and are highly hopeful of reaching that of native blood vessels, as listed in Table 2. For example, in Table 2, the saphenous vein’s longitudinal elastic modulus (stiffness) can be 130 or 23.7 MPa. The mean diameter of the usual great saphenous vein (GSV) is 5.0 ± 2.4 mm. The mean diameter of a typical small saphenous vein (SSV) is 3.1 ± 1.3 mm [314].

Regarding the dimensions, various polymers and corresponding fabrication skills presented in Table 1 can meet the requirement, such as silicone, PU, heparin-releasing PLLA/PCL, and PEG/collagen/PU. For the stiffness, 10% (*w*/*v*) P(CL/LA)/PGLA (sealed) and 15% (*w*/*v*) P(CL/LA)/PGLA (sealed) are capable of matching the stiffness of native small saphenous with 23.7 MPa [312]. However, most synthetic polymers’ stiffness lies in the range of kPa. Apart from that, polymers’ suture retention strength and burst pressure are still predominantly lower than native vessels. Compared to single synthetic-polymer-made scaffolds, a mixture of polymers with or without biological molecules/natural polymers demonstrated potential neovascularization ability [273]. Therefore, new challenges arise in this field, and these issues are becoming more specific and detailed. How do we control the components’ percentage of composite polymers to optimize biomechanical properties? How can we choose suitable polymer partners among hundreds of polymer families? The methods and choices are increasing, but at the same time, the complexity of studies and characterization of those synthetic polymer-based scaffolds are also being augmented. Similar to vascular replacements created by natural polymers, more exploration and studies should be conducted and established to develop acellular vessel prostheses with small- and macro-dimensions.

More importantly, the future perspective for developing synthetic-polymer scaffolds should focus on enhancing biophysical performance, such as neovascularization. Some vascular scaffolds proved insufficient for cell regrowth due to the porosity and fabrication techniques used [315]. As an example, vascular scaffolds developed from electrospinning have been shown to possess low capacities for cell migration, adhesion, viability, and proliferation [316]. The relatively small pore sizes support these facts within electrospun scaffolds. Small pore sizes prevent cell infiltration and metabolite, nutrients, and waste diffusion. Synthetic (polyurethane and PLGA) and natural (derived from gelatin) polymers used to create electrospun scaffolds adversely influenced cell bioactivities due to their pore size and porosity [317]. Besides, cytotoxic solvents used in forming scaffolds’ surfaces, inferior structural integrities, and limited degradation rates have been shown to inhibit vascular remodeling and recellularization [318]. As a result, the successful creation of synthetic polymeric vascular tubes demands paying attention to the properties of polymers and other easily ignorable influencers, such as the cytotoxic solvents and agents residual in tissue-engineering technologies.

## 6. Conclusions and Proposed Models of Polymeric Vascular Substitutes

**Figure 3:**
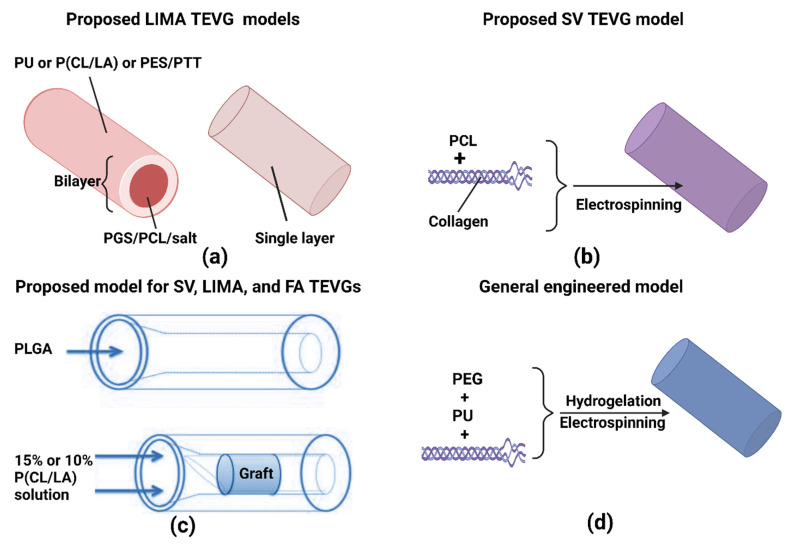
Proposed models for polymeric TEVGs. (**a**) Proposed LIMA TECG models, (**b**) proposed SV TEVG model (**c**) proposed model for SV, LIMA, and FA TEVGs, and (**d**) general engineered model.

Acellular TEVGs have been developed strikingly throughout the years with profound potential geared towards multiscale blood vessels. Nevertheless, it cannot be denied that critical challenges still exist [315]. Thus, there is a definite need to enhance polymeric replacements’ physical and biomechanical properties and optimize their safety and effectiveness for final clinical transformation. Therefore, we proposed a few models of TEVGs (Figure 3) composed of polymers based on characterization and known requirements (Table 1) to assist in the future creation of polymeric TEVGs.

From Table 2, there are three types of blood vessels, which are saphenous veins (SVs), left internal mammary arteries (LIMAs), and the femoral artery (FA). The mean diameter of the great saphenous vein (GSV) is 5.0 ± 2.4 mm. The mean diameter of an average small saphenous vein (SSV) is 3.1 ± 1.3 mm [314]. Thus, the dimension range of SV is 1.8–7.4 mm. The diameter of LIMA ranges from 0.8 mm to 3.0 mm [319,320], while that of FA is 2.5–9.6 mm [321]. However, LIMAs are either micro- or small-scale, and the other two cross the micro, small, and macro scales. For the LIMA model, we suggest that the scaffold consisting of PGS, PCL, and salt be cast and molded, referring to Table 2. The elastic modulus (EM) of scaffolds with the same components is around 0.5 MPa, which is way lower than native LIMAs. As a result, the model should have one more layer fabricated by PU, P(CL/LA), or polyester (PES)/PTT or should consider the combination of these polymers with high EM (Figure 3a). 

The bilayer model might be created first through casting/molding, then electrospinning. For SVs that require the EM and UTS to be 4.2 MPa and 1.8 MPa, respectively [107,190]. a tubular scaffold composed of PCL and collagen can be achieved via electrospinning (Figure 3b). In addition, the acellular model works for all three types of blood vessels based on either 10% or 15% (*w*/*v*) of P(CL/LA)/PGLA. Composites with 15% (*w*/*v*) of P(CL/LA)/PGLA are more favored when the EM and UTS are increased [281]. This method dissolved a 40:60 copolymer sealant solution of P(CL/LA) (Sigma-Aldrich, St. Louis, MO, USA) in 1,4-dioxane at 10% and 15% *w*/*v* ratios and then was cast and electrospun [281]. The final model (Figure 3c) are PGLA-P(CL/LA) vascular grafts sealed with 10% P(CL/LA) and 15% P(CL/LA). Since the EM and UTS will not change with the diameter size, this model contains PGLA and P(CL/LA) and could be conducted based on multiple dimensions in theory [281]. According to those mentioned above, the generally engineered vascular analogs made by polymers should have the following features: the burst pressure should be at least 260 kPa, and the EM should at least be 100–200 kPa [230,322]. For engineered capillaries, the maximum thickness should be 200 μm [322]. In this case, in order to meet at least two requirements, burst pressure (BP) and EM, except for the models mentioned above, PEG/collagen/PU vascular scaffolds with the small ID generated by electrospinning and hydrogelation can be referred to as a future example (Figure 3d).

In conclusion, the models proposed for TEVGs formed by polymers are generic models that meet at least two requirements in correspondence with biomechanical characterization. However, further studies are needed to improve vascular prostheses’ physical and biological performances. These studies include but are not limited to the relevant components’ percentile of the admixture of various polymers; efficient and effective fabrication techniques; the simulation of the mechanical and surface heterogeneities within the microenvironment of native vessel segments and engineered scaffolds, especially multilayered structures [323,324]; and down-reaching explorations of macro-scale polymeric TEVGs.

## Data Availability

Not applicable.
